# Genetic etiology and pregnancy outcomes of fetuses with central nervous system anomalies

**DOI:** 10.1007/s00404-023-07152-z

**Published:** 2023-07-21

**Authors:** Huimin Tao, Jiebin Wu, Yu Han, Bei Zhang, Jingfang Zhai

**Affiliations:** 1 Xuzhou Clinical College of Xuzhou Medical University, Xuzhou, 221000 Jiangsu China; 2 Department of Prenatal Diagnosis Medical Center, Xuzhou Central Hospital, Xuzhou, China; 3 Key Laboratory of Brain Diseases Bioinformation, Xuzhou Medical University, Xuzhou, China

**Keywords:** Central nervous system anomalies, Fetus, Pregnancy management, Copy number variant sequencing, Dynamic ultrasound screening

## Abstract

**Purpose:**

To investigate genetic etiology and pregnancy outcomes of fetal central nervous system (CNS) anomalies.

**Methods:**

217 fetuses with CNS anomalies were included in our cohort from January 2016 to December 2022. 124 cases received karyotyping and 73 cases simultaneously underwent copy number variant sequencing (CNV-seq). Dynamic ultrasound screening and pregnancy outcomes were followed up, including neonates’ neurodevelopmental outcomes.

**Results:**

(1) 20 types of CNS anomalies were revealed by ultrasound and the most common was ventriculomegaly. (2) 14 (11.3%) of 124 cases were found chromosomal abnormalities by karyotyping, and copy number variations (CNVs) were revealed in 13 (17.8%) of 73 cases by CNV-seq. Fetuses with non-isolated CNS anomalies had a higher detection rate (DR) of abnormal karyotypes and CNVs than those with isolated CNS anomalies (25.0% vs. 4.8%; 35.0% vs. 11.3%) (*P* < 0.05). And the DR of abnormal karyotypes was significantly higher in multiple CNS anomalies than in single CNS anomaly (16.7% vs. 2.8%, *P* < 0.05), while there were no significant differences in the DR of CNVs. (3) Through dynamic ultrasound, 12 cases were further found progression or additional malformations. (4) Pregnancy outcomes of 209 cases were obtained, including 136 (65.1%) live births, 3 (1.4%) intrauterine fetal deaths, and 70 (33.5%) terminated. Two neonatal deaths at 6 months and one infant with motor and intellectual disabilities were finally found after long-term follow-up.

**Conclusion:**

Genetic analysis combined with dynamic ultrasound screening and multidisciplinary consultation plays an important role in evaluating the prognosis of fetal CNS anomalies, especially for those with multiple CNS or extracranial abnormalities.

## What does this study add to the clinical work

**Table:** 

Genetic analysis contributed to evaluating the prognosis of fetuses with CNS anomalies. Especially with multiple CNS or extracranial abnormalities. Their pregnancy outcomes also provided a basis for clinicians to better manage the affected fetuses.	

## Introduction

Fetal central nervous system (CNS) anomalies are one of the most common congenital malformations in fetuses, such as ventriculomegaly and hydrocephalus, with an incidence of 1% [1]. Genetic factor plays a significant role in the occurrence of CNS anomalies [2], and invasive prenatal diagnosis is recommended to exclude genetic abnormalities. Compared with traditional karyotype analysis, copy number variation sequencing (CNV-seq) can not only detect chromosomal abnormalities, but also reveal additional copy number variations (CNVs) [3]. It also has advantages of higher throughput, whole genome coverage and lower cost over chromosomal microarray analysis [4]. A combination of CNV-seq and karyotyping performed in fetal structural malformations could improve the detection rate (DR) of chromosomal aberrations [5]. To the day, there has been few literatures using the two techniques in the detection of fetal CNS anomalies.

However, it’s more challenging for clinicians to assess the outcomes and manage fetuses with CNS anomalies, as fetal brain development is a continuous process throughout pregnancy. Hence, it’s significant to comprehensively evaluate the affected fetuses through genetic analysis, dynamic ultrasound examination and multidisciplinary consultation [6], which helps to ensure the best possible outcomes for mother and fetus [7]. In our study, we systematically investigated the results of prenatal diagnosis and pregnancy outcomes of fetal CNS anomalies in order to provide a basis for better prenatal counseling and management.

## Materials and methods

This retrospective study was performed in the prenatal diagnosis center of Xuzhou Central Hospital from January 2016 to December 2022. 217 fetuses with CNS anomalies detected by ultrasonography were included in our cohort. 124 cases received invasive prenatal diagnosis, including 77 amniocentesis and 47 cordocentesis. All cases were successfully karyotyped and 73 cases underwent CNV-seq simultaneously.

A comprehensive assessment of fetal CNS structures was conducted according to relevant guidelines [8, 9]. Dynamic ultrasound follow-ups were conducted for further assessments. All cases were divided into two groups: isolated CNS anomalies and non-isolated CNS anomalies when complicated with both CNS and extra-CNS anomalies. If two or more CNS anomalies were revealed, isolated CNS anomalies were further classified as single or multiple CNS anomalies.

This study has been approved by the Ethics Committee of Xuzhou Central Hospital (XZXY-LK-20230314-035). All the couples provided informed consents before genetic testing and received clinical counseling.

### Karyotype analysis

Fetal samples were obtained by ultrasound-guided amniocentesis or cordocentesis in terms of gestational weeks. Amniotic fluid or umbilical cord blood samples were cultured, harvested, prepared, G-banded, and analyzed. All karyotyping reports were interpreted according to the International System for Human Cytogenetic Nomenclature at the level of 300–400 bands (ISCN 2016/ISCN 2020).

### CNV-seq and data analysis

Fetal genomic DNA was extracted from amniotic fluid or umbilical cord blood, and DNA libraries were constructed as the manufacturer’s instructions. CNV-seq was then carried out using the NextSeq550AR platform (Illumina, USA) for massively parallel sequencing (0.1x). Sequencing data were analyzed by GISTIC 2.0 and annotated to the human reference genome sequence version GRCh37/hg19. The results were interpreted based on the following databases: Database of Genomic Variants (DGV, http://dgv.tcag.ca/dgv/), Online Mendelian Inheritance in Man (OMIM, http://www.ncbi.nlm.nih.gov/omim), Chromosomal Imbalance and Phenotype in Humans Using Ensemble Resources (DECIPHER, https://decipher.sanger.ac.uk/) and guidelines of the American College of Medical Genetics (ACMG) [10]. Benign/Likely Benign CNVs were not included in our statistic. Parental peripheral blood samples were suggested to be further tested for fetuses with CNVs.

### Follow-up of pregnancy outcomes

A multidisciplinary team (MDT) including departments of ultrasound, prenatal diagnosis and pediatric neurology comprehensively evaluated the prognosis of fetuses with CNS anomalies to aid the parents in decision-making. Pregnancy outcomes were followed up via postpartum telephone and electronic medical record system. Survivors’ neurodevelopmental outcomes were assessed by pediatricians from 6 months to 2 years in our study.

### Statistical analyses

SPSS 26.0 software (IBM Corp., Armonk, NY, USA) was used for statistical analyses. Count data were expressed as percentages. The chi-square test was used to compare the rates between groups, and *P* < 0.05 was regarded as statistically significant.

## Results

### General characteristics of fetal CNS anomalies

A total of 217 fetuses with CNS anomalies were enrolled in the study. The average maternal age was 28.64 ± 4.68 (18–43) years, and the mean gestational age was 26.56 ± 13.54 (12^+2^–38^+5^) weeks. There were 79 (36.4%) primigravida, and among the remaining 138 (63.6%) pregnant women, eight cases had a history of adverse pregnancy including three terminated due to CNS abnormalities, two congenital heart diseases, one cervical hygroma, one pathogenic CNV, and one infant death for microcephaly.

20 types of CNS anomalies were revealed by prenatal ultrasound in Table 1. The most common was ventriculomegaly (35.8%), followed by widened posterior fossa (WPF) (15.7%), and choroid plexus cyst (9.7%). 217 fetuses with CNS anomalies were divided into 162 isolated CNS anomalies (classified as 136 single anomaly and 26 multiple anomalies), and 55 non-isolated CNS anomalies. In the latter group, cardiovascular system anomalies (35.1%, 27/77) ranked first, other extra-CNS malformations included urinary system (15.6%, 12/77), facial malformations (13.0%, 10/77), gastrointestinal malformations (7.8%, 6/77), umbilical abnormalities (7.8%, 6/77), polyhydramnios or oligohydramnios (6.5%, 5/77), extremities (3.9%, 3/77), hepatobiliary (2.6%, 2/77) and others. Subependymal cyst often occurred in isolation, whereas vermis hypoplasia, Dandy-Walker malformation (DWM), holoprosencephaly and agenesis of corpus callosum (ACC) tended to be associated with multiple structural malformations.

**Table 1:** Distributions of CNS anomalies in 217 fetuses

Types of CNS anomalies	Isolated CNS anomalies		Non-isolated CNS anomalies	Total (*n*, %)
	Single	Multiple		
*Ventricular abnormalities*				
Ventriculomegaly	58 (42.6)	16 (27.6)	22 (29.7)	96 (35.8)
Hydrocephalus	8 (5.9)	4 (6.9)	1 (1.4)	13 (4.9)
*Posterior fossa abnormalities*				
WPF	25 (18.4)	10 (17.2)	7 (9.5)	42 (15.7)
Vermis hypoplasia	0 (0)	2 (3.4)	2 (2.7)	4 (1.5)
Blake cyst	3 (2.2)	2 (3.4)	1 (1.4)	6 (2.2)
DWM	1 (0.7)	0 (0)	3 (4.1)	4 (1.5)
*Midbrain abnormalities*				
Holoprosencephaly	3 (2.2)	1 (1.7)	10 (13.5)	14 (5.2)
ACC	1 (0.7)	8 (13.8)	4 (5.4)	13 (4.9)
*Neural tube defects*				
Spina bifida	3 (2.2)	0 (0)	1 (1.4)	4 (1.5)
Anencephaly	1 (0.7)	0 (0)	1 (1.4)	2 (0.7)
Exencephaly	1 (0.7)	0 (0)	1 (1.4)	2 (0.7)
*Choroid plexus cyst*	13 (9.6)	4 (6.9)	9 (12.2)	26 (9.7)
*Subependymal cyst*	9 (6.6)	0 (0)	3 (4.1)	12 (4.5)
*Arachnoid cyst*	9 (6.6)	4 (6.9)	4 (5.4)	17 (6.3)
*Absent CSP*	0 (0)	5 (8.6)	2 (2.7)	7 (2.6)
*Others*^a^	1 (0.7)	2 (3.4)	3 (4.1)	6 (2.2)

*WPF* widened posterior fossa, *DWM* Dandy-Walker malformation, *ACC* agenesis of corpus callosum, *CSP* cavum septi pellucidi

^a^Schizencephaly (*n* = 2), microcephaly (*n* = 1), intracranial hemorrhage (*n* = 1), hydranencephaly (*n* = 1) and dural sinus malformation (*n* = 1)

### Karyotyping results of 124 fetuses with CNS anomalies

14 (11.3%) of 124 cases were found to have abnormal karyotypes (Tables 2 and 3). Trisomy 21 (*n* = 3) and trisomy 18 (*n* = 3) were the most common chromosomal abnormalities. Other numerical abnormalities (*n* = 3) included 45,X, mos47, XXY[21]/46, XY[79], mos46, XX[43]/46, XY[57], structural abnormalities (*n* = 5) included 46,XX,del(5)(q14), 46,XX,del(1)(p36), 46,XY,inv(9)(p12q21.1), 46,XX,der(6)t(6;10)(p25;p13), 46,XY,der(16)t(7;16)(p14.1;p13.3). The DR of abnormal karyotypes was significantly higher in multiple CNS anomalies than in single CNS anomaly (16.7% vs. 2.8%, χ^2^ = 4.375, *P* = 0.036). Meanwhile, fetuses with non-isolated CNS anomalies had a higher DR of abnormal karyotypes than those with isolated CNS anomalies (25.0% vs. 4.8%, χ^2^ = 11.081, *P* = 0.001), suggesting that fetuses with multiple CNS or extra-CNS anomalies were more likely to have chromosomal abnormalities.

**Table 2:** Distributions of abnormal karyotypes in 124 fetuses with CNS anomalies

Karyotyping	Total	Isolated CNS anomalies			Non-isolated CNS anomalies
		Single	Multiple	*n*	
Number	124	72	12	84	40
Normal karyotype	110	70	10	80	30
Abnormal karyotype (*n*, %)	14 (11.3)	2 (2.8)	2 (16.7)	4 (4.8)	10 (25.0)
χ^2^		4.375		11.081	
*P*		0.036		0.001

**Table 3:** Abnormal karyotypes in 14 fetuses with CNS anomalies

Number	Ultrasound findings	Karyotyping
1	WPF; Thickened nuchal fold	45,X
2	Holoprosencephaly; Cleft lip and palate; Enlarged gallbladder; CHD	47,XY, + 18
3	Holoprosencephaly; Facial deformity; CHD	47,XY, + 18
4	ACC; Choroid plexus cyst; Arachnoid cyst; CHD	47,XY, + 18
5	Ventriculomegaly; Echogenic bowel	47,XY, + 21
6	Ventriculomegaly; Thickened nuchal fold; Ventricular bright spot	47,XX, + 21
7	Ventriculomegaly; Nasal bone dysplasia; VSD	47,XX, + 21
8	Ventriculomegaly; Arachnoid cyst	46,XX,del(5)(q14)
9	Ventriculomegaly; WPF; CHD	46,XX,del(1)(p36)
10	Ventriculomegaly	46,XY,inv(9)(p12q21.1)
11	Ventriculomegaly	mos47,XXY[21]/46,XY[79]
12	Hydrocephalus; ACC	mos46,XX[43]/46,XY[57]
13	Arachnoid cyst; Bilateral renal pelvis separation	46,XX,der(6)t(6;10)(p25;p13)
14	Vermis hypoplasia; Cleft lip and palate; Micrognathia; CHD; Strephenopodia with polydactyly	46,XY,der(16)t(7;16)(p14.1;p13.3)

*WPF* widened posterior fossa, *CHD* congenital heart disease, *ACC* agenesis of corpus callosum, *VSD* ventricular septal defect

### CNV-seq results of 73 fetuses with CNS anomalies

Chromosomal aberrations were revealed in 13 (17.8%) of 73 cases by CNV-seq (Tables 4 and 5). Except that 5 cases were consistent with the results of karyotype analysis (size > 5 Mb), the remaining 8 (11.0%) cases were additionally detected by CNV-seq. The DR of pathogenic/likely pathogenic (P/LP) CNVs and variants of uncertain significance (VUS) were 11.0% (8/73) and 6.8% (5/73), respectively. Of the 8 cases with P/LP CNVs, 6 cases were known as microdeletion/microduplication syndromes, including 1p36 microdeletion syndrome, 16p11.2 microdeletion syndrome, Bosch-Boonstra-Schaaf optic atrophy syndrome (BBSOAS), Axenfeld-Rieger syndrome type 3 (RIEG3), ATR-16 syndrome and Simpson-Golabi-Behmel Syndrome Type 1 (SGBS1). Fetuses with non-isolated CNS anomalies had a higher DR of CNVs than those with isolated CNS anomalies (35.0% vs. 11.3%, χ^2^ = 5.562, *P* = 0.018), indicating that the incidence of CNVs increased when combined with extracranial malformations. However, no significant differences were found between single and multiple CNS anomalies (9.3% vs. 20.0%, χ^2^ = 0.925, *P* = 0.336).

**Table 4:** Distributions of CNVs detected by CNV-seq

CNV-seq	Total	Isolated CNS anomalies			Non-isolated CNS anomalies
		Single	Multiple	*n*	
Number	73	43	10	53	20
Normal	60	39	8	47	13
CNVs (*n*, %)	13 (17.8)	4 (9.3)	2 (20.0)	6 (11.3)	7 (35.0)
P/LP CNVs (*n*, %)	8 (11.0)	1 (2.3)	1 (10.0)	2 (3.8)	6 (30.0)
VUS (*n*, %)	5 (6.8)	3 (7.0)	1 (10.0)	4 (7.5)	1 (5.0)
Additional CNVs (*n*, %)	8 (11.0)	4 (9.3)	1 (10.0)	5 (9.4)	3 (15.0)
χ^2^		0.925		5.562	
*P*		0.336		0.018

**Table 5:** P/LP and VUS CNVs in 13 fetuses with CNS anomalies

Number	Ultrasound findings	CNVs	Size (Mb)	Rating	Related syndromes	Inheritance	Outcome
1	Ventriculomegaly; Arachnoid cyst; Double renal pelvis	del(1)(p36.32p36.21)	2.80	VUS	–	Maternal	Delivery
2	Ventriculomegaly; Absent CSP	dup(6)(q23.3)	1.40	VUS	–	*	TOP
3	Ventriculomegaly	del(16)(p13.11)	0.78	VUS	16p13.11 recurrent region	*	TOP
4	Ventriculomegaly	dup(X)(p22.12)	1.10	VUS	–	Maternal	Delivery
5	WPF	dup(X)(p22.31p22.32)	1.70	VUS	Xp22.31 recurrent region	*	Delivery
6	Ventriculomegaly; Strephenopodia	del(16)(p11.2)	0.48	LP	16p11.2 microdeletion syndrome	Paternal	TOP
7	Ventriculomegaly; WPF; CHD	del(1)(p36.33p36.22)	8.70	P	1p36 microdeletion syndrome	*	TOP
8	Ventriculomegaly; Arachnoid cyst	del(5)(q14.3q15)	7.94	P	BBSOAS	*	TOP
9	Arachnoid cyst; Bilateral renal pelvis separation	del(6)(p25.3p25.1) dup(10)(p15.3p13)	6.32 13.94	P VUS	RIEG3	*	TOP
10	Vermis hypoplasia; Cleft lip and palate; Micrognathia; CHD; Strephenopodia with polydactyly	del(16)(p13.3p13.3) dup(7)(p22.3p14.1)	1.46 37.50	P P	ATR-16 syndrome	Paternal	TOP
11	Ventriculomegaly	del(X)(p22.13p22.12)	1.00	P	–	de novo	TOP
12	DWM; VSD; Thickened nuchal fold; Single umbilical artery; Acromphalus	del(X)(q26.2q26.2)	0.26	P	SGBS1	de novo	TOP
13	Ventriculomegaly; Nasal bone dysplasia; VSD	dup(21)(q11.1q22.3)	33.83	P	–	*	TOP

*CSP* cavum septi pellucidi, *WPF* widened posterior fossa, *CHD* congenital heart disease, *DWM* Dandy-Walker malformation, *VSD* ventricular septal defect, *TOP* termination of pregnancy

*No further testing

### Follow-up in 209 fetuses with CNS anomalies

With the involvement of MDT, pregnancy outcomes of 209 cases were obtained, including 136 (65.1%) live births, 3 (1.4%) intrauterine fetal deaths (IUFD) and 70 (33.5%) terminations (Table 6). Pregnancies were terminated due to abnormal karyotypes (*n* = 10, including 5 P/LP CNVs), P/LP CNVs (*n* = 3), and VUS (*n* = 2: one case with a 0.78-Mb 16p13.11 deletion progressed from mild ventriculomegaly to hydrocephalus, and one case with a 1.40-Mb 6q23.3 deletion was further found ACC). In addition, 36 fetuses with severe CNS malformations and 18 fetuses with severe multiple malformations accounted for termination of pregnancy (TOP). During dynamic ultrasound follow-ups, 12 cases with progression or additional structural malformations were finally found (4 cases mild ventriculomegaly turned to hydrocephalus, 4 cases were further detected with ACC, 2 cases with holoprosencephaly, 1 case with DWM, and 1 case with VSD).

**Table 6:** Pregnancy outcomes in fetuses with different CNS anomalies

Pregnancy outcomes	Live birth	TOP	IUFD
*Ventricular abnormalities*			
Ventriculomegaly	67	25	
Hydrocephalus	1	11	1
*Posterior fossa abnormalities*			
WPF	31	8	
Vermis hypoplasia		4	
Blake cyst	4	2	
DWM		4	
*Midbrain abnormalities*			
Holoprosencephaly		13	1
ACC	3	9	1
*Neural tube defects*			
Spina bifida		4	
Anencephaly		2	
Exencephaly		2	
*Choroid plexus cyst*	22	3	
*Subependymal cyst*	10	1	
*Arachnoid cyst*	11	5	
*Absent CSP*	1	6	
*Others*^a^		6	

*TOP* termination of pregnancy, *IUFD* intrauterine fetal death

^a^Schizencephaly (*n* = 2), microcephaly (*n* = 1), intracranial hemorrhage (*n *= 1), hydranencephaly (*n* = 1) and dural sinus malformation (*n* = 1)

After long-term follow-up from 6 months to 2 years after delivery, 112 out of 136 neonates’ outcomes could be obtained. Two neonatal deaths at 6 months were found as follows: one showed WPF, arachnoid cyst, Blake cyst with normal karyotype, and the other one manifested as ventriculomegaly and ACC without genetic testing. In addition, one infant without chromosomal aberrations was found to have both motor and intellectual disabilities presenting with ventriculomegaly, absent CSP, ACC and left ventricular hyperecho by prenatal ultrasound. The remaining 109 infants were alive without any significant abnormalities detected, including three cases with VUS CNVs (two cases were inherited from their mothers and one was not verified by parents). They were prenatally evaluated to have relatively good outcomes.

## Discussion

CNS anomalies are associated with poor outcomes such as motor delay, mental disability and even death in children [11]. Chromosomal numerical and structural abnormalities were the earliest identified factors leading to fetal CNS anomalies, accounting for 8.0% [2, 12]. In our study, the overall DR of chromosomal abnormalities was 11.3% (14/124) by karyotyping. Trisomy 21 and trisomy 18 were the most common aneuploidies, consistent with the previous reports [6, 12]. Our study showed that the DR of abnormal karyotypes was significantly higher in multiple CNS anomalies than that in single CNS anomaly, and higher in non-isolated CNS anomalies than in isolated CNS anomalies (Table 2), which indicated that multiple CNS or extracranial malformations might increase the risk of genetic abnormalities [13, 14]. We also found that 7 of 14 cases with abnormal karyotypes were detected with ventriculomegaly, mostly related to trisomy 21. 7 cases were complicated with cardiovascular system abnormalities, and 7 cases presented with more than two kinds of structural abnormalities (Table 3). Therefore, more attention should be paid to fetuses with ventriculomegaly, and invasive prenatal diagnosis should be recommended for the occurrence of multiple structural malformations, especially cardiovascular system.

CNVs occurred in 16.4% of fetuses with CNS anomalies [12], similar to our study, CNV-seq detected 13 (17.8%) abnormal CNVs, with an additional DR of 11.0% (Table 4), including some genetic syndromes such as 16p11.2 microdeletion syndrome, BBSOAS and 1p36 microdeletion syndrome. It indicated that fetal CNS anomalies were associated with chromosomal microdeletions or microduplications, which can be detected by CNV-seq to make up for the shortcomings of traditional karyotyping [3, 5]. Therefore, CNV-seq together with karyotyping should be recommended for fetal CNS anomalies to clarify genetic etiology and provide scientific proof for prenatal counseling. Consistent with previous findings [14, 15], the DR of CNVs in non-isolated CNS anomalies was significantly higher than that in isolated CNS anomalies, which suggested that the risk of CNVs increased when extra-CNS abnormalities were found. However, there was no statistical significance between single and multiple CNS anomalies. Therefore, CNV-seq is particularly suggested for fetuses with multiple or non-isolated CNS anomalies because of relatively higher incidences of CNVs. Moreover, 9 of 13 cases with CNVs were detected with ventriculomegaly (Table 5), which indicates that ventriculomegaly was associated with both chromosome aneuploidies and CNVs [16]. Therefore, genetic testing is strongly recommended for fetuses with ventriculomegaly to identify the cause.

Prenatal ultrasound is a useful tool for screening fetal structural malformations, but it has the possibility of under/overdiagnosis due to the limitations of fetal position, gestational week, amniotic fluid volume, etc. In our study, a total of 20 different types of CNS anomalies were revealed, and ventriculomegaly accounted for the highest proportion. Vermis hypoplasia, DWM, holoprosencephaly and ACC were more likely to be associated with multiple structural abnormalities, thus careful examination for both CNS and extra-CNS structures should be performed, especially fetal cardiovascular system. The routine second trimester scan may fail to find some CNS anomalies occurring later in pregnancy, such as intracranial hemorrhage, hydrocephaly and tumors [17]. Through dynamic ultrasound follow-ups in our study, 12 cases were further detected with progression or additional structural abnormalities and terminated ultimately. Therefore, dynamic sonographic assessments are crucial to confirm the diagnosis and evaluate the prognosis of fetuses with CNS abnormalities, which might alter the pregnancy outcomes [18]. However, as the prognosis of the affected fetuses is often complex, a multidisciplinary team should be recommended to assess the possible prognosis and provide better perinatal management. Joint discussion among clinicians and families helps to fully inform the parents of the current condition and make decisions as soon as possible [19]. Meanwhile, CNV-seq detection of the parents to verify whether the detected fetal CNVs were inherited or de novo is also helpful to evaluate the prognosis of the fetus.

When facing fetal CNS anomalies, the parents are usually unprepared and their decisions can be affected by various factors. In our study, TOP was chosen in 70 cases for chromosomal abnormalities, severe CNS or multiple structural malformations (Table 6). Whether to continue the pregnancy may be influenced by genetic etiology, the type and complexity of neurological malformations, and involvement of extra-CNS anomalies. In addition, maternal age, gestational age, and presence of children can influence the choice [20]. Although some parents make up their minds to continue the pregnancy, the fetus may suffer spontaneous death [20, 21]. In our study, three in-utero fetal demises were found due to multiple CNS and compound extracranial anomalies. However, more data is needed to confirm the correlation between multiple structural abnormalities and higher rates of IUFD. Even with good prenatal prediction, fetuses with CNS abnormalities may also have unpredictable deaths and poor neurodevelopmental outcomes, which can be seen in our study that two neonatal died at 6 months and one infant were detected with neurodevelopmentally abnormal. From Table 6, when subependymal cyst, choroid plexus cyst or Blake cyst was detected without other abnormalities, dynamic ultrasound follow-up was preferentially recommended to parents due to high incidence of live births. However, for ventriculomegaly, holoprosencephaly and ACC, further genetic testing should be recommended because they are more likely to be associated with chromosomal abnormalities (Tables 3 and 5). Furthermore, postpartum assessments should also be offered to survivors for better long-term management of such children.

There are still some limitations in our study. In the cases of induced labor, autopsy can be a convincing examination to verify the results of ultrasound screening and provide beneficial suggestions for the next pregnancy [22]. However, only two families received the pathological examination in our study. What’s more, only half of the cases received invasive prenatal diagnosis, which bring clinicians more troubles in assessing their genetic background and future prognosis.

In conclusion, fetal CNS anomalies is related to chromosome aneuploidies and CNVs, especially for those with multiple or non-isolated CNS abnormalities. Genetic analysis combined with dynamic ultrasound screening and multidisciplinary consultation plays an important role in evaluating the prognosis of fetuses with CNS anomalies.

## Data Availability

The data in the study are available on request from the corresponding author, Jingfang Zhai, upon reasonable request.
